# Modeling Volcanic Eruption Parameters by Near-Source Internal Gravity Waves

**DOI:** 10.1038/srep36727

**Published:** 2016-11-10

**Authors:** M. Ripepe, G. Barfucci, S. De Angelis, D. Delle Donne, G. Lacanna, E. Marchetti

**Affiliations:** 1 Dipartimento Scienze della Terra, Università di Firenze, via LaPira, 4, 50121 Firenze, Italy; 2 School of Ocean and Earth Sciences, J. Herdman Building, University of Liverpool, 4 Brownlow Street, L69 3GP, Liverpool, UK; 3 Dipartimento di Scienze della Terra e del Mare, Università degli Studi di Palermo, Palermo, Italy

## Abstract

Volcanic explosions release large amounts of hot gas and ash into the atmosphere to form plumes rising several kilometers above eruptive vents, which can pose serious risk on human health and aviation also at several thousands of kilometers from the volcanic source. However the most sophisticate atmospheric models and eruptive plume dynamics require input parameters such as duration of the ejection phase and total mass erupted to constrain the quantity of ash dispersed in the atmosphere and to efficiently evaluate the related hazard. The sudden ejection of this large quantity of ash can perturb the equilibrium of the whole atmosphere triggering oscillations well below the frequencies of acoustic waves, down to much longer periods typical of gravity waves. We show that atmospheric gravity oscillations induced by volcanic eruptions and recorded by pressure sensors can be modeled as a compact source representing the rate of erupted volcanic mass. We demonstrate the feasibility of using gravity waves to derive eruption source parameters such as duration of the injection and total erupted mass with direct application in constraining plume and ash dispersal models.

Volcanoes are powerful source of infrasound capable of perturbing the atmosphere well below the frequencies of acoustic waves, down to longer periods typical of gravity waves^1^,^2^,^3^,^4^,^5^,^6^,^7^,^8^,^9^. Gravity waves arise from perturbations of the hydrostatic equilibrium initiated by the displacement of the fluid medium and can propagate at the free surface of a fluid, or at the interface between two fluids with different densities, or within a medium with internal density stratification. During eruptions, the atmosphere is displaced by the sudden explosive ejection of a large amount of hot gas and volcanic ash. In a stable atmosphere the force of gravity will act to restore equilibrium, thus, generating oscillations called internal gravity waves.

Observations of gravity waves associated with volcanic activity are still rare, mainly limited to large sub-plinian events, and frequently recorded at large distance from the source (>100 km). Acoustic-gravity waves with periods of about 300 seconds (3.3 mHz) but propagating at near-sound speed of 210–320 m/s have been recorded by seismometers and microbarometers during large plinian eruptions, including Mount St. Helens in 1980^1^,^2^, El Chichon in 1982^3^,^4^, and Mount Pinatubo in 1991^4^,^5^,^6^,^7^. These oscillations were modeled as the response of the atmosphere to either mass or energy injection during the eruption^2^,^7^. However, thus far, such models have only been validated with data recorded at regional or global (i.e. hundreds to thousands of km) distances from the source. More recently, gravity waves generated by vulcanian explosions at Soufrière Hills Volcano (SHV) on Montserrat (WI), were recorded at significantly smaller distance (<7 km) from the source by microbarometers and differential pressure sensors^8^,^9^ providing a unique opportunity to test models using observations at near-source (<10 km) distance^9^.

Here we exploit data collected by four stations at SHV to analyze in detail propagation and waveform of gravity waves in the near-source region where distance to the source is smaller than their wavelength^7^. We show that a simple point source model^2^,^7^ allows constraining the location of the source and to model the source time function in terms of mass eruption rate providing reliable total erupted volumes. Our results suggest that continued observations of large-scale atmospheric perturbations may have future application in assessing the relative magnitude of volcanic explosions and will contribute to improve our knowledge on the input source parameters for accurate ash dispersal models.

## Results

Soufrière Hills Volcano is an andesitic lava dome volcano located at the Northern end of the Lesser Antilles island arc. Its eruptive activity over the past two decades has been characterized by the cyclic growth and collapse of a summit dome punctuated by moderate-to-violent vulcanian explosions, producing ash plumes with heights of up to 20 km before collapsing to form pumice fountains feeding large pyroclastic flows^10^,^11^.

**Figure 1:**
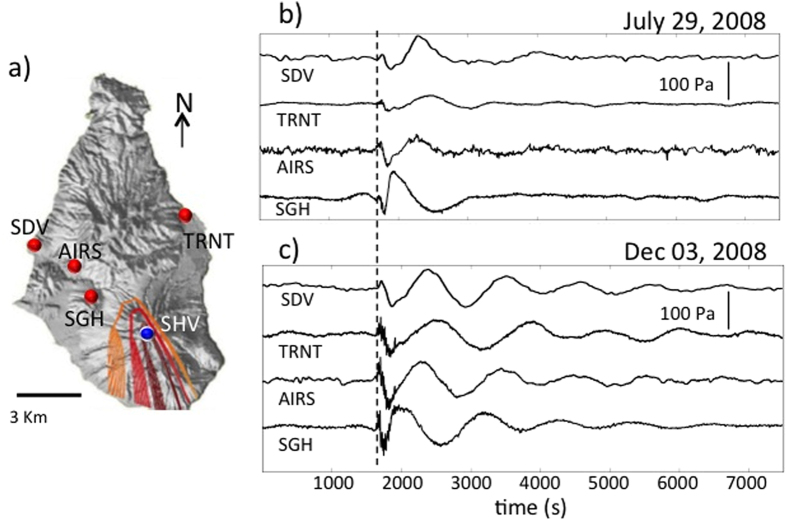
(**a**) Location of the microbarographs located at St. George’s Hill (SGH), Air Studios (AIRS), Trants Bay (TRNT), and Old Town (SDV). The contour lines represent the limit of confidence of the location of the acoustic source. Values >0.8 indicate the most probable location of the source and coincide with the SHV dome. Original ultra-low pressure signals recorded at the 4 stations and relative to the July 29, 2008 (**b**) and December 03, 2008 (**c**) vulcanian explosions. The dashed line indicates the onset of the vulcanian eruptions which were at 03:38:13 GMT on July 29, 2008 and at 01:37:15 on December 3, 2008.

In March 2008 a permanent small-aperture infrasonic array was installed by the Department of Earth Sciences (DST), University of Florence, Italy in collaboration with the Montserrat Volcano Observatory. The array, located at a distance of ~3 km from the summit of SHV, at the St. George’s Hill site (SGH), has demonstrated its ability to detect and locate explosive events and pyroclastic flows in real-time^8^,^12^. The central station of the array is equipped with two differential pressure transducers with sensitivity of 25 mV/Pa in the frequency band 0.01–50 Hz, and self-noise of 10^−2^ Pa. Atmospheric pressure perturbations are additionally monitored by microbarometers deployed on Montserrat with the aim to correct for long-period atmospheric disturbances in gravimetric and strain signals. The locations of microbarometers (Fig. 1a) include Old Town (SDV), Air Studios (AIRS), and Trants Bay (TRNT). The gravimetric station SDV, located ~6.8 km NW of the active vent, is equipped with a Vasaila Digital Barometer PTB210 with a resolution of −3 nm/s2/hPa, and data are recorded with a sampling rate of 0.1 Hz^13^. Two strainmeters installed within the framework of the CALIPSO borehole observatory^14^ at the sites AIRS, 4.7 km from the vent, and TRNT, 6.6 km from the vent, are equipped with SETRA microbarometers, sampled at 1 Hz with 20 bits resolution^9^.

**Figure 2:**
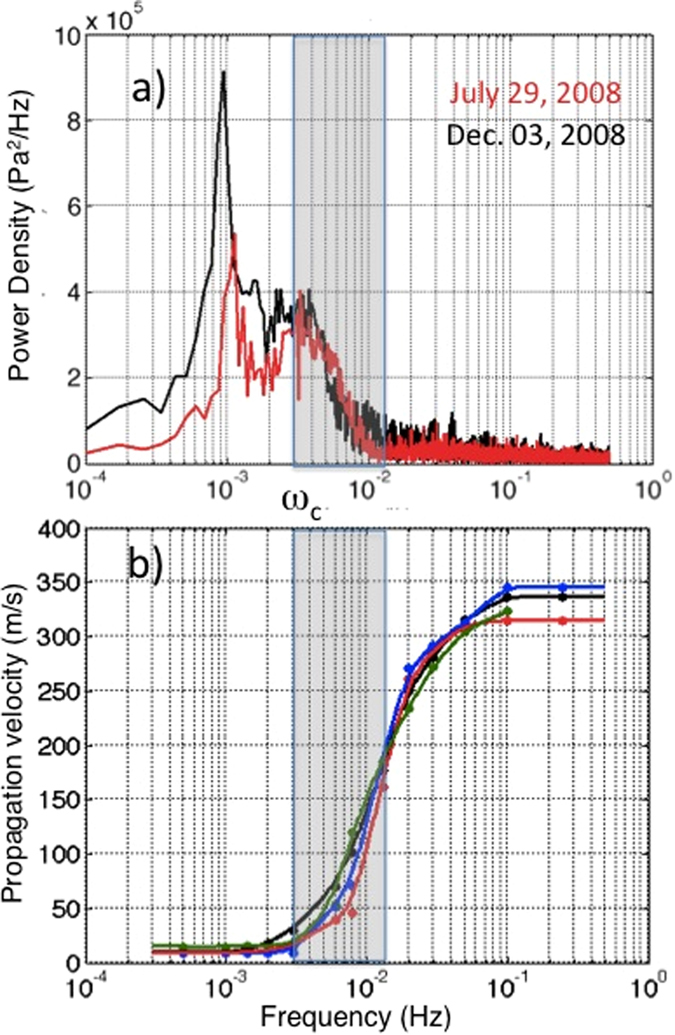
(**a**) Spectral content of the acoustic signals recorded at the SGH during the July 29 (red line) and the December 03, 2008 (black line) vulcanian explosions show a large energy at frequencies of ~1 mHz typical of gravity waves. Power Density Spectrum for the July pressure signal (red line) has been multiplied by a factor of 5. (**b**) Propagation velocity of the acoustic signal calculated in different frequency bands from 1 mHz to 0.3 Hz and for different combination of stations (blue line SGH-TRNT; black line AIRS-TRNT; red line SGH-AIRS; green line SDV-AIRS) shows that frequencies below the cut-off frequency (*ω*_c_) are traveling at a speed between 8 and 15 m/s. Velocity increases up to 340 m/s for frequencies >0.01 Hz. The gray band indicates part of the spectrum, which shows interaction between gravity waves and pure acoustic waves.

The pressure sensors and microbarometers recorded large-scale atmospheric pressure perturbations associated with two vulcanian explosions at SHV on July 29 and December 3, 2008 (Fig. 1b,c). These events occurred during the fourth extrusive phase of the 1995-present eruption of SHV^15^ and produced ash plumes with a height of about 12 km^16^. Once corrected for the instrument response, all sensors showed an ultra low-frequency signal with a duration in excess of 4000 s associated with the explosive activity (Fig. 1b,c). The spectra of these ultra long-period oscillations show peaks at frequencies of 0.97 and 1.1 mHz (Fig. 2a), well below the threshold of acoustic waves and consistent with the frequencies of atmospheric gravity waves^8^,^9^,^16^. Here we analyze and model the propagation and the waveform of these gravity waves.

### Source Location and Propagation Speed

**Figure 3:**
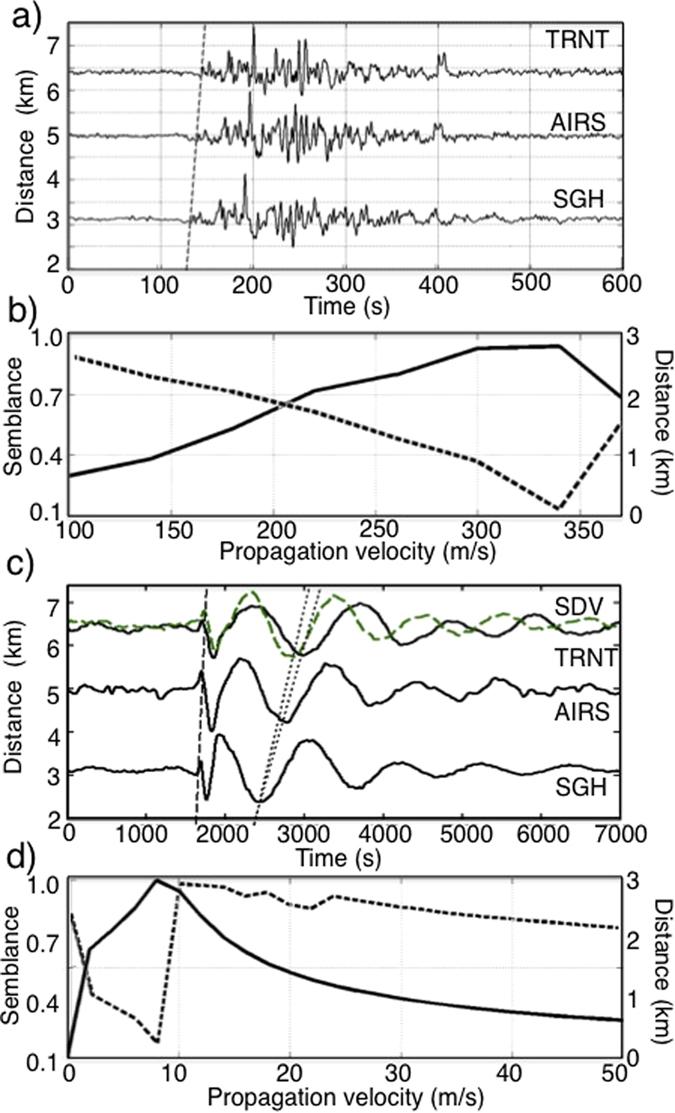
(**a**) Pressure signal associated with the vulcanian explosion of December 03, 2008 and recorded at the three stations (SGH, TRNT, AIRS) shows coherent waveforms when is filtered in the acoustic range between 0.01 and 0.5 Hz. The results of the grid searching procedure for the two different frequency band are represented in terms of propagation velocity as function of semblance (bold line) and distance from the dome (dashed line). We found that for the acoustic waves (**b**) the best solution has semblance (black line) of 0.99 for a propagation velocity of 344 m/s which corresponds to the minimum distance (dashed line) of 394 m from the SHV dome. (**c**) The same pressure signal low-pass filtered <0.003 Hz evidence ultra-low frequency content typical of gravity waves. (**d**) Using the same grid searching procedure we found that the best solution has a semblance (black line) of 0.90 for a propagation velocity of 8 m/s which gives the minimum distance (dashed line) from the SHV dome of 590 m. This demonstrates that also the ultra-low frequency is generated above the volcanic dome.

The instrument-corrected pressure signals were initially filtered in the infrasonic frequency band between 0.01 and 0.4 Hz (Fig. 3a) and a grid search procedure was applied to locate the acoustic source. This procedure assumes that the source could be located at each node of the 9200 × 8700 m grid (Fig. 1a) with node spacing of 100 m and located at ~910 m a.s.l., corresponding to the elevation of the SHV crater rim. For each node in the grid, theoretical source-receiver travel-times are calculated for sound velocities between 100 and 400 m/s. The acoustic signals are delayed for the theoretical source-receiver travel time, and multichannel semblance analysis^17^ is used to identify the best source location. The highest semblance of 0.99 corresponds to a velocity of 344 m/s (Fig. 3b bold line) compatible with the sound speed in the atmosphere at ambient condition and air temperature of 22 °C. The location of the infrasonic source is 394 m (Fig. 3b dashed line) southwest of the crater rim (Fig. 1a) and is consistent with the area were numerous vents were active in particular during December 2008.

The same grid and node spacing was used to search for the best location of the source of the pressure signal band-pass filtered in the frequency range of gravity waves around 1 mHz (Fig. 3c). In this case, velocity was allowed to vary between 5 and 50 m/s and the highest semblance of 0.9 resulted from a propagation velocity of 8 m/s (Fig. 3d bold line). Such a low propagation velocity is consistent with the observed arrival move-out across the network (Fig. 3c) and is common for gravity waves^18^. Unfortunately, at such low propagation velocities and frequency content, source location is not accurately resolved as in the case of acoustic waves and it extends over a relatively large area from the dome 590 m (Fig. 3d dashed line) towards the South.

### Propagation of Acoustic and Gravity Waves

We used the source position to assess the dispersion characteristics along the source-receiver path. We assumed that each frequency of the spectrum represents a wave packet generated at the same time by the same source.

The pressure signals recorded at each station were analyzed in different frequency bands between 1 mHz and 0.25 Hz measuring the phase shift between pairs of station, which converted in phase velocities (Fig. 2b) assuming a stable source position for each frequency component. In agreement with source location, at frequencies above 0.02 Hz pressure waves propagates at 300–335 m/s the phase velocity of the sound waves (Fig. 3b), whilst at frequencies below 3 mHz the pressure perturbation travels with velocities between 8 and 15 m/s (Fig. 3d), typical of internal gravity waves^19^,^20^. Phase velocity increases from 40 m/s up to 300 m/s in the frequency range between 3 mHz and 20 mHz. This analysis demonstrates that pressure waves of different nature are generated by the same explosive source and exhibit an inverse dispersive behaviour with frequency-dependent propagation velocity.

Lamb’s theory demonstrates that in an isothermal, and stratified, atmosphere with mean temperature of 290 K and mean molecular mass of 4.76 × 10^−26^ kg (78% N and 22% O), acoustic waves do not propagate below the acoustic cutoff frequency:


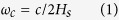


of 0.019 rad/s (~3 mHz), which is defined as the ratio between the mean speed of sound *c* (325 m/s) in the troposphere, and the atmospheric scale height *H*_*s*_ of 8.6 km, calculated for the temperature profile of the atmosphere above SHV.

This suggests that acoustic waves generated by volcanic explosions can, theoretically, only propagate at frequencies >3.0 mHz. Our dispersion analysis shows that, gravity waves with velocities <15 m/s are observed (Fig. 2b) below this threshold and pure acoustic waves propagating at the speed of sound (>325 m/s) are detected only at frequencies greater than 0.02 Hz (Fig. 2b). Further, this analysis shows that in the frequency range between 3 and 20 mHz the propagation velocity of the pressure perturbation assumes intermediate apparent velocity (20–120 m/s) making it difficult to separate gravity from acoustic waves.

### Near-field Propagation of Gravity Waves

The propagation of gravity waves is described by the two-dimensional Euler equations for an irrotational and inviscid flow under the Boussinesq approximation, known as the Taylor-Goldstein equation^20^. Considering no background velocity field (i.e. null wind speed), the solution of the Taylor-Goldstein equation leads to a dispersion relation for the angular frequency *ω*_c_ in the form:


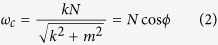


where Φ is the angle between the wave vector and the horizontal direction, *k* and *m* are the horizontal and vertical wavenumber, respectively. The *Brunt-Väisälä* frequency *N* of the atmosphere is the angular frequency at which a fluid parcel, vertically displaced from its equilibrium position, oscillates within a stable stratified fluid and is related to the thermal stratification of the atmosphere defined in terms of potential temperature^20^.

The dispersion relation of equation (2) shows that for waves propagating horizontally Φ = 0 (fluid parcel displaced vertically), their frequency *ω*_*x*_ will coincide with the *Brunt-Väisälä* frequency (ω_*x*_ = *N*). Using the atmospheric profile measured by a radiosonde and available twice a day (at 12 AM and 12 PM) at one of the Global Observing System (GOS) stations (TFFR, 78897), in Guadeloupe, (located at 80 km from Montserrat) we calculated from the vertical temperature and pressure profiles the *Brunt-Väisälä* frequency, *N*, on July 29 and December 3, 2008. Within the troposphere, up to an elevation of ~15 km, *N* fluctuates around mean values of 0.011 (on July 29) and 0.010 rad s^−1^ (on Dec. 03) increasing to 0.02 rad s^−1^ in the stratosphere. These values correspond to frequencies above 1.6 mHz and higher than what measured during both eruptions.

Internal gravity waves propagating at an angle from the horizontal *ϕ* ≠ 0 have frequency between 0 and *N* (0 < *ω*_*x*_ < *N*) and their phase velocity in the horizontal direction *c*_*x*_ is:


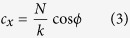


Considering the *Brunt-Väisälä* and the observed frequencies (0.97 and 1.1 mHz) for the gravity waves at SHV, we calculate from equation (3) angles *ϕ* of 48.9°and 52.4° for the two eruptions. At a distance *x* of ~5000 m from the vent, this suggests a source located at an elevation, *z*_*s*_ = *x* tan (90−*ϕ*) between 3800 and 4300 m a.s.l. Slight variations in the frequency content of gravity waves during the two events may result from the combination of changes in atmospheric conditions and variations in the source position.

### Gravity Waves and Eruption Source Parameters

The sudden expansion of the eruptive gas-and-ash cloud is the responsible for displacing the atmosphere, and thus, generating gravity waves (Figs 1b,c and 3c) with peak amplitudes of 16.8 and 34 Pa, during the July and December 2008 events, respectively. Following classical point source theory^2^,^7^,^21^,^22^, the resulting oscillations of the free atmosphere *h*(*x, t*) can be modeled as induced by a unit mass injection point source represented by a step function^7^:


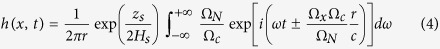


located at elevation *z*_*s*_ above the flat ground and at a slant distance *r* = (*z*_*s*_^2^ + *x*^2^)^1/2^ from the sensor, and where 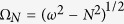, 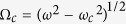 and 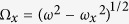 are calculated assuming the atmospheric profile at the time of the eruptions. To avoid possible contamination induced by the filtering, the integral in equation (4) has been numerically solved in the same frequency range *ω*_*x*_ ≤ *ω* ≤ *ω*_*c*_ of the observed gravity waves and it represents the response of the atmosphere in m^−1^ to the unitary step of the mass flow rate^7^.

**Figure 4:**
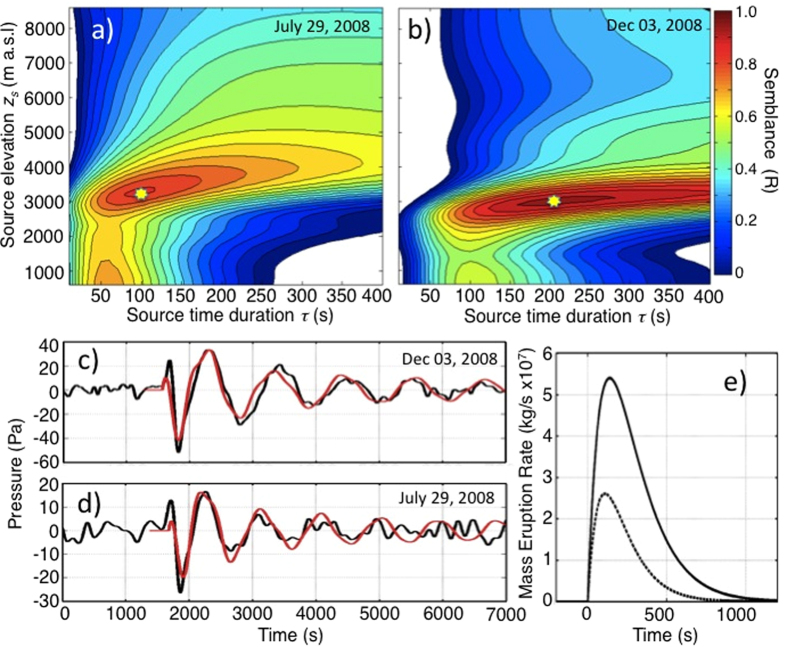
Forward modeling the gravity waveforms using the equations (4) and (5). The best source time function (equation 5) in searched for a source time duration, *τ*, between 10 and 400 s (in steps of 10 s) and a source elevation between 600 and 9000 m (in steps of 100 m). The best solution (yellow star) is represented by the maximum semblance between the measured and the normalized modeled waveform. Color bar indicates the semblance between the modeled and the measured gravity wave. (**a**) For the July 29 eruption the best fit of R = 0.86 is represented by a source located at an elevation of 3300 m a.s.l. above the ground and a duration *τ* = 100 s of the mass injection. (**b**) For the December 03 explosion, the best fit (R = 0.96) is obtained for a source located at an elevation of 3200 m above the ground and for a duration *τ* = 190 s of the source function. Comparison between the gravity waves recorded (black lines) during (**c**) the December 03 and (**d**) the July 29, 2008, vulcanian explosions and the pressure calculated using the point mass injection source model (red lines) using equation (9) and forward searching algorithm. (**e**) Mass eruption rates used as source function (equation (5)) to model the gravity waves associated with the July 29 (dashed line) and the December 03, 2008, (bold line) explosions.

We assume that the mass flow rate, *q*(*t*), can be represented by the exponential source function^2^,^7^:


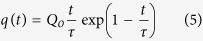


where *τ* is the duration of the source function in seconds, and *Q*_*o*_ is the peak mass eruption rate in kg s^−1^ (Fig. 4e). Pressure changes *P*(*x, t*) induced on the ground by the gravity waves and recorded at a distance *x* from the source can be, thus, modeled as the convolution *P*(*x, t*) = *q*(*t*)*h(*x, t*) between the oscillation of the free atmosphere *h*(*x, t*) and the first time derivative of the mass ejection rate *q*(*t*).

The best solution for our forward modeling is represented by the minimum misfit between the recorded signal and the modeled *P*(*x, t*) searched for a source duration (*τ*) in the range between 10 and 400 s (steps of 10 s) and a source elevation (*z*_*s*_) between 600 and 9000 m (steps of 100 m) (Fig. 4a,b). The best fit with the recorded gravity waves (semblance 0.86) is obtained, for the July 29 eruption (Fig. 4d), by a source located at an elevation of 3300 m a.s.l. and with a duration *τ* = 100 s of the mass injection (Fig. 4a). The peak pressure of 16.8 Pa gives a peak mass eruption rate *Q*_*o*_ = 2.7 × 10^7^ kg/s (Fig. 4e). In the case of the December 03 explosion, the best fit (semblance 0.96) between the model and the observed gravity wave (Fig. 4c) is obtained for a source located at an elevation of 3200 m above the ground and for a source duration *τ* = 190 s (Fig. 4b). In this second case, the peak pressure of 34.6 Pa requires a mass eruption rate of *Q*_*o*_ = 5.4 × 10^7^ kg/s (Fig. 4e).

The fit between the modeled and the observed gravity waveforms is impressive and it allows us to derive additional parameters from the source time function. Integration over time of the modeled source mass eruption rate, *q*(*t*), gives a total erupted mass of 0.7 × 10^10^ kg and of 2.0 × 10^10^ kg, for the July and December explosions, respectively. These values convert to a total DRE volume of 2.5 × 10^6^ m^3^ and 7.6 × 10^6^ m^3^ for the two events. For the July explosion, the duration of the modeled source function (*τ* = 100 s) is very close to the duration of 105 s estimated from strain data^23^ whereas the mass eruption rate derived from gravity waves (2.7 × 10^7^ kg/s) is remarkably similar to the 2.3 × 10^7^ kg/s estimated from tephra deposit^16^.

## Discussion

Mass discharge rate and duration of the mass injection phase represents the key parameters to decipher the dynamics of volcanic eruption, which have always been difficult to assess with sufficient accuracy. The violent injection of a large amount of a gas and ash mixture can excite the atmosphere inducing acoustic-gravity modes which have been recorded by seismometers^3^,^4^,^5^,^18^,^24^ and microbarometers^2^,^6^,^7^,^8^,^9^. However, in the near field, gravity waves do not couple to the ground efficiently and are not easy to be observed on seismograms^18^. We show how the low propagation velocity (<10 m/s) makes these waves detectable using low-cost small-aperture infrasonic arrays frequently deployed on volcanoes to monitor explosive activity at local distances (<10 km).

Gravity waves have also been observed in satellite imagery^7^,^25^ as expanding pressure fronts centered around the volcano and consistent with the free oscillation of a fluid isothermal atmosphere triggered by the point injection of a large mass of volcanic material^2^,^7^. We have shown how the point source model^2^,^7^,^21^,^22^ provides a reasonable and simple physical solution to explain the origin of atmospheric gravity oscillations at ~1 mHz also in near-source region^7^,^9^.

Position of the source indicates the height where the initial momentum of the ejected material is exhausted^26^ and our modeling is suggesting that this elevation is reached at around 3200 m a.s.l (that is at ~2286 m above the SHV crater). The duration of the source time function shows that the initial momentum sustained the eruptive column at SHV for ~100–200 s by continuous ejection of ash and gas mixture, feeding ~10^10^ kg of tephra in the atmosphere. The total erupted mass derived from the forward modeling of the gravity waves (0.7 × 10^10^ kg and of 2.0 × 10^10^ kg) can be used to estimate the possible height of the umbrella column. Considering the empirical relationship *H* = 0.041*M*^0.25^ for the strong plume and assuming no wind effect^27^, we calculate that each explosion reached the 12 and 15 km of elevation, respectively, which are in very good agreement with the observed plume height^16^.

The analysis of atmospheric gravity waves generated by volcanic explosions and recorded also near the source by microbarometers can thus provide quantitative information on the size, rate and duration of eruptions. Simple parameters such as duration of the ejection phase and total mass erupted are critical inputs for atmospheric ash dispersal models. We show that these vital parameters can be derived by modeling gravity waves in near real-time. The results presented in this work may have important implications in the assessment of volcanic activity and provide a framework for the interpretation of atmospheric oscillation excited by volcanic eruption.

## Additional Information

**How to cite this article**: Ripepe, M. *et al*. Modeling Volcanic Eruption Parameters by Near-Source Internal Gravity Waves. *Sci. Rep*. **6**, 36727; doi: 10.1038/srep36727 (2016).

**Publisher’s note:** Springer Nature remains neutral with regard to jurisdictional claims in published maps and institutional affiliations.
